# Communication Complexity of Entanglement-Assisted Multi-Party Computation

**DOI:** 10.3390/e26110896

**Published:** 2024-10-23

**Authors:** Ruoyu Meng, Aditya Ramamoorthy

**Affiliations:** Department of Electrical and Computer Engineering, Iowa State University, Ames, IA 50011, USA; adityar@iastate.edu

**Keywords:** communication complexity, entanglement-assisted communication, integer linear programming

## Abstract

We consider a quantum and a classical version of a multi-party function computation problem with *n* players, where players 2,…,n need to communicate appropriate information to player 1 so that a “generalized” inner product function with an appropriate promise can be calculated. In the quantum version of the protocol, the players have access to entangled qudits but the communication is still classical. The communication complexity of a given protocol is the total number of classical bits that need to be communicated. When *n* is prime, and for our chosen function, we exhibit a quantum protocol (with complexity (n−1)(logn) bits) and a classical protocol (with complexity $({\left(n-1\right)}^{2}\left(\log n^{2}\right)$ bits)). Furthermore, we present an integer linear programming formulation for determining a lower bound on the classical communication complexity. This demonstrates that our quantum protocol is strictly better than classical protocols.

## 1. Introduction

We consider a multi-party function computation scenario in this work. There are a total of *n* players in the system numbered 1,2,…,n. Each player observes her input and players 2,…,n (remote parties) communicate an appropriate number of bits that allows player 1 to finally compute the value of the function. Clearly, this can be accomplished if players 2,…,n communicate their actual values, but in many cases, the function value can be computed with much less information. Thus, a key question is to determine the minimum number of bits the remote parties need to send to player 1.

Such problems are broadly studied under the umbrella of communication complexity [1,2] in the literature. In this work, we consider the zero-error version of this problem. Our main goal is to understand the advantage that the availability of quantum entanglement confers on this problem and compare it with classical protocols. Such problems have a long history in the literature [3,4].

**Background:** There are three kinds of quantum protocols within quantum communication complexity (QCC) problems. In the first kind (introduced by Yao [2]), each player communicates via a quantum channel and the metric is the number of qubits transmitted. We call it the quantum transmission model. The second variation assumes that each player can use entanglement as a free resource, but the communication remains classical; the primary metric here is the number of classical bits transmitted. We refer to this as the entanglement model, which was introduced by Cleve and Buhrman [5]. The third kind is a combination of the first two. We call it the combined model. It allows free usage of entanglement and works with quantum communication. The work of de Wolf [6] shows that, in the two-party case, the entanglement model can be reduced to the quantum transmission model with a two-fold penalty, utilizing teleportation [7].

Buhrman, Cleve, Wigderson [8] and Cleve, van Dam, Nielsen, and Tapp [9] considered the case of the two-party function computation with quantum communication and used reduction techniques to connect problems in QCC to other known problems, and derived upper/lower bounds for QCC in this manner. In particular, the first work [8] showed examples, such as the set disjointness function, where quantum protocols are strictly better than classical ones in the bounded-error setting. Here, the set-disjointness problem is such that each player has a set and wants to decide if their intersection is empty. Buhrman and de Wolf [10] generalized the two-party "log-rank" lower bound of classical communication complexity to QCC where quantum protocols use both shared entanglement and quantum communication. For other two-party upper/lower bound techniques, see [11,12,13,14,15].

**Related Work:** We will now discuss works in multi-party quantum communication complexity. There are mainly two kinds of models. The number-in-hand (NIH) model assumes each player observes only one variable. The number-on-forehead (NOF) model assumes each player observes all but one variable. François and Shogo [16] considered the NIH model with quantum communication and provided a quantum protocol for a three-party triangle-finding problem; the formulation considers bounded error. This has a polynomial advantage with respect to any classical protocol. Here, the triangle-finding problem is such that the edge set of a graph is distributed over each user and the task is to find a triangle of the graph.

The results in the following two works apply to both NIH and NOF models. Lee, Schechtman, and Shraibman [17] proved a Grothendieck-type inequality and subsequently derived a general lower bound for the multi-party QCC for the Boolean function in Yao’s model. Following this work, Briet, Buhrman, Lee, and Vidick [18] showed a similar inequality for the multi-party XOR game and established that the discrepancy method provides lower bounds for QCC when the combined model is of the third type discussed earlier.

Buhrman, van Dam, Høyer, and Tapp [19] considered the NIH model with shared entanglement and proposed a three-party problem with a quantum protocol that is better than any classical protocol by a constant factor. Following this work, Xue, Li, Zhang, Guo [20], and Galv ao [21] showed similar results under the same function with more restrictions. The work most closely related to ours is by Cleve and Buhrman [5]. This study involved three players (Alice, Bob, and Carol) who each have *m*-bit strings, denoted as $\vec{x},\vec{y}$, and $\vec{z}$, respectively. The strings are such that $\vec{x}+\vec{y}+\vec{z}=1$, meaning their binary sum (modulo-2) results in the all-ones vector. The goal is for Alice to compute the following:

$$\begin{array}{cc} g(x,y,z) & =\sum_{i=1}^{m}x_{i}y_{i}z_{i} \end{array}$$

using binary arithmetic. We note that the communication from Bob and Carol to Alice is purely classical; however, they can use entanglement in a judicious manner. For this particular function, ref. [5] shows that a classical protocol (without entanglement) requires three bits of communication, whereas if the parties share 3m entangled qubits, then two bits of communication are sufficient.

**Main Contributions:** In this work, we consider a generalization of the original work of [5]. In particular, we consider a scenario with *n* players (for prime *n*) that observe values that lie in a higher-order finite field, with a more general promise that is satisfied by the observed values. As we consider more players and higher-order finite fields, the techniques used in the original work are not directly applicable in our setting. For instance, when n=3, our generalized inner product function is defined over $F_{3}$ arithmetic (modulo-3), whereas in the same setting, ref. [5] considers binary (modulo-2) arithmetic. Thus, even though we consider a similar problem, we highlight that the result of [5] cannot be recovered as a special case of our result.

Our work provides the following contributions:

- We demonstrate a quantum protocol that allows for the function to be computed with (n−1)logn bits. We use the quantum Fourier transform as a key ingredient in our method.
- On the other hand, we demonstrate a classical protocol that requires the communication of ${\left(n-1\right)}^{2}\left(\log n^{2}\right)$ bits.
- To obtain a lower bound on the classical communication complexity, we define an appropriate integer linear programming problem that demonstrates that our quantum protocol is strictly better than any classical protocol.

This paper is organized as follows. Section 2 discusses the problem formulation and Section 3 discusses our quantum protocol. Section 4 and Section 5 discuss our classical protocol and the lower bound on any classical protocol, respectively.

## 2. Problem Formulation

### 2.1. Classical/Quantum Communication Scenarios

Let $X_{i},i=1,\ldots ,n$ and Y denote the sets in which the inputs and the output lie, and let $f\left(x_{1},\ldots ,x_{n}\right):X_{1}\times \cdots \times X_{n}\mapsto Y$ be a multivariate function. There are *n* players, and the *i*-th player is given $x_{i}\in X_{i}$. The first player (henceforth, Alice) receives information from each of the players; this communication should allow her to compute $f(x_{1},\ldots ,x_{n})$. The players are not allowed to communicate with each other.

In the classical protocol, players 2 to *n* communicate to Alice via classical channels. In the quantum protocol, we assume that the users have access to shared entanglement as a free resource; however, the communication remains classical. In both scenarios, the classical/quantum communication complexity is defined as the minimum number of classical bits transmitted such that Alice can compute the function among all classical/quantum protocols.

### 2.2. Generalized Inner Product Function with a Promise

In this work, we consider a specific multivariate function and the setting where n≥3 (number of players) is prime. Let $F_{n}$ denote the finite field of order-*n* and [m]≜{1,…,m}. The *i*-th player is given a vector ${\vec{x}}^{i}={\left[x_{1}^{i}\ldots x_{m}^{i}\right]}^{T}\in F_{n}^{m}$, i.e., each $x_{j}^{i}\in F_{n}$. The vectors satisfy the following “promise”: ∀j∈[m], the *j*-th component of each player’s vector is such that we have the following:

$$\begin{array}{cc} {\left[x_{j}^{1},\ldots ,x_{j}^{n}\right]}^{T}\in & \{a{\left[1,\ldots ,1\right]}^{T}+b{\left[0,\ldots ,n-1\right]}^{T}\left|a,b\in F_{n}\right\}, \end{array}$$

i.e., ${\left[x_{j}^{1},\ldots ,x_{j}^{n}\right]}^{T}$ lies in a two-dimensional vector space spanned by the basis vectors ${\left[1,\ldots ,1\right]}^{T}$ and ${\left[0,1,\ldots ,n-1\right]}^{T}$. In this case, it can be observed that ${\left[x_{j}^{1},\ldots ,x_{j}^{n}\right]}^{T}$ is either a multiple of the all-ones vector (if b=0) or a permutation of [0,1,…,n−1] (if b≠0). The function to be computed is the generalized inner product function given by the following:

(1) $$\begin{array}{c} GIP\left({\vec{x}}^{1},\ldots ,{\vec{x}}^{n}\right)=\sum_{i=1}^{m}\left(\prod_{j=1}^{n}x_{i}^{j}\right), \end{array}$$

where the operations are over $F_{n}$.

**Remark** **1.** *In [5], the promise equivalently means that the j-th component of Alice, Bob, and Carol’s vector lies in a two-dimensional affine subspace over $F_{2}$ given by $\{a\cdot \vec{v_{1}}+b\cdot \vec{v_{2}}\in F_{2}^{3}:a,b\in F_{2}\}+1.$ Thus, the promise introduced in our work can be considered as a natural higher-order generalization of their promise.*

## 3. Proposed Quantum Protocol

We first discuss the entangled states and unitary transforms that will be used in the proposed quantum protocol in Section 3.1. In Section 3.2, we discuss the quantum protocol in detail, including a proof of its correctness. A word about notations: In the following, for complex vectors $\vec{u},\vec{v}$, $\langle \vec{u},\vec{v}\rangle =\sum_{i}u_{i}^{†}v_{i}$ denotes the usual inner product. On the other hand, if $\vec{u},\vec{v}\in F_{n}^{m}$, then $\langle \vec{u},\vec{v}\rangle =\sum_{i=1}^{m}u_{i}v_{i}$ denotes the inner product over $F_{n}$. Moreover, $\delta_{ij}$ denotes the Kronecker delta function, which equals 1 if i=j and 0 otherwise. All logarithms in this paper are to base-2.

### 3.1. Entanglement Resource and Unitary Transforms Used

#### 3.1.1. Shared Entangled States

Consider *n* isomorphic *n*-dimensional quantum systems, where each system has a computational basis denoted B={|0〉,|1〉,…,|n−1〉}. There are *m* entangled states shared among *n* players. For i∈[m], we prepare the entangled state as follows:

(2) $$\begin{array}{c} |\Phi_{i}\rangle :=\frac{1}{\sqrt{n}}\sum_{k=0}^{n-1}|k\ldots k\rangle . \end{array}$$

The *j*-th subsystem of this entangled state is given to *j*-th player for j=1,…,n.

#### 3.1.2. Quantum Fourier Transform

Let $\omega :=e^{\frac{2\pi i}{n}}$ denote the *n*-th root of unity. The quantum Fourier transform (QFT) is defined by the following unitary map:

(3) $$|j\rangle \mapsto \frac{1}{\sqrt{n}}\sum_{k=0}^{n-1}\omega^{jk}|k\rangle ,\forall |j\rangle \in B.$$

Let $QFT^{⊗l}$ denote the QFT performed over *l* isomorphic systems.

#### 3.1.3. Phase Shift Map

For $j\in F_{n}$, we define the following:

$$\begin{array}{cc} \; & P_{0}≜\left\{\begin{array}{c} |0\rangle \mapsto \omega^{-\frac{n-1}{2}}|0\rangle \\ |i\rangle \mapsto |i\rangle ,i\neq 0. \end{array}\right. \end{array}$$

(4) $$\begin{array}{cc} \mathrm{If}\;j\neq 0, & P_{j}≜|i\rangle \mapsto \omega^{-\frac{1}{n}\left(ij\;\mathrm{mod}\;n\right)}|i\rangle . \end{array}$$

### 3.2. The Quantum Protocol

Next, we introduce the quantum protocol that uses (n−1)(logn) bits.

**Theorem** **1.** *There exists a quantum protocol for computing $GIP({\vec{x}}^{1},\ldots ,{\vec{x}}^{n})$, which uses (n−1)logn bits.*

In our protocol (see Algorithm 1), for each i=1,…,m, each player *p* examines $x_{i}^{p}$ and applies the corresponding phase shift map to her subsystem of $|\Phi_{i}\rangle$. Following this, she applies the QFT on each of her symbols and then measures in the computational basis; this yields $s_{i}^{p}\in F_{n}$ for i=1,…,m. Player *p* then transmits $\sum_{i=1}^{m}s_{i}^{p}$. As the players 2≤p≤n transmit a symbol from $F_{n}$, it is clear that the total communication in the protocol is (n−1)(logn).
**Algorithm 1:** Proposed quantum protocol.For i∈{1,…,m}, prepare maximally entangled “shared state” $|\Phi_{i}\rangle$ (cf. (2)) and distribute corresponding subsystems to all players.**for** player p∈{1,…n} **do**    **for** each i∈{1,…,m} **do**        Assume $x_{i}^{p}=j$, then player *p* applies $P_{j}$ (cf. (4)) on her part of $|\Phi_{i}\rangle$.        Player *p* performs QFT on her part of the shared state.        Player *p* measures her part of the shared state in the computational basis, yielding $s_{i}^{p}\in F_{n}$    **end for**    $s^{p}\leftarrow \sum_{i=1}^{m}s_{i}^{p}$    Player *p* sends $s^{p}$ to Alice if p≠1**end for**$GIP\left({\vec{x}}^{1},\ldots ,{\vec{x}}^{n}\right)=\sum_{p}s^{p}.$

To show the proof of correctness of the protocol, we need the following auxiliary lemma. The proof appears in Appendix A:

**Lemma** **1.** *Let $\vec{\alpha }={\left[1,\ldots ,1\right]}^{T}\in F_{n}^{n}$. Then, for each $x\in F_{n}$, we have the following:*

(5) $$\begin{array}{c} QFT^{⊗n}\left(\frac{1}{\sqrt{n}}\sum_{j=0}^{n-1}\omega^{-jx}|j\cdot \vec{\alpha }\rangle \right)=\frac{1}{n^{\frac{n}{2}}}\sum_{\vec{k}\in {\left\{0,\ldots ,n-1\right\}}^{n}}a_{\vec{k}}|\vec{k}\rangle . \end{array}$$

*Then the amplitude $a_{\vec{k}}\neq 0$ iff $\sum_{j=1}^{n}k_{j}=x$ where $\vec{k}={\left[k_{1},\ldots ,k_{n}\right]}^{T}$.*

The proof of correctness of the protocol hinges on the following lemma:

**Lemma** **2.**
(6) $$\sum_{p=1}^{n}s_{i}^{p}=\prod_{p=1}^{n}x_{i}^{p},\mathrm{for}\;i=1,\ldots ,m.$$

**Proof.**  The state jointly measured by each player is as follows:

$$QFT^{⊗n}\left(\sum_{j=0}^{n-1}\left({⊗}_{p=1}^{n}P_{x_{i}^{p}}\right)\frac{1}{\sqrt{n}}|j\cdot \vec{\alpha }\rangle \right),$$

where $\vec{\alpha }={\left[1,\ldots ,1\right]}^{T}$. If ${\left[x_{i}^{1},\ldots ,x_{i}^{n}\right]}^{T}={\left[j,\ldots ,j\right]}^{T}$, then we have the following (see Appendix B for derivation):

(7) $$\begin{array}{c} P_{j}^{⊗n}\left(\frac{1}{\sqrt{n}}\sum_{k=0}^{n-1}|k\cdot \vec{\alpha }\rangle \right)\mapsto \frac{1}{\sqrt{n}}\sum_{k=0}^{n-1}\omega^{-kj}|k\cdot \vec{\alpha }\rangle . \end{array}$$

Thus, $QFT^{⊗n}\left(\frac{1}{\sqrt{n}}\sum_{k=0}^{n-1}\omega^{-kj}|k\cdot \vec{\alpha }\rangle \right)$ has non-zero coefficients only for states $|\vec{k}\rangle$ such that $\sum_{l=1}^{n}k_{l}=j$ by Lemma 1. Therefore, the measurement result ${\left[s_{i}^{1},\ldots ,s_{i}^{n}\right]}^{T}$ must be one of $\vec{k}={\left[k_{1},\ldots ,k_{n}\right]}^{T}$ s.t.

$$\sum_{l=1}^{n}k_{l}=j\overset{\left(a\right)}{=}j^{n}=\prod_{p=1}^{n}x_{i}^{p}$$

where (a) follows from the fact that $j\in F_{n}$.

Now, we assume the following: ${\left[x_{j}^{1},\ldots ,x_{j}^{n}\right]}^{T}=a{\left[1,\ldots ,1\right]}^{T}+b{\left[0,1,\ldots ,n-1\right]}^{T}$ with b≠0. This implies that $a+b\cdot i\in F_{n}$ is distinct for each i∈{0,…,n}. It can be observed that a[1,…,1]+b[0,…,n−1] is a permutation of [0,…,n−1], so it suffices to discuss ${\left[x_{i}^{1},\ldots ,x_{i}^{p}\right]}^{T}={\left[0,\ldots ,n-1\right]}^{T}$ by symmetry. We have the following (see Appendix B for derivation):

(8) $$\begin{array}{cc} & P_{0}⊗\cdots ⊗P_{n-1}\left(\frac{1}{\sqrt{n}}\sum_{k=0}^{n-1}|k\cdot \vec{\alpha }\rangle \right)\mapsto \frac{1}{\sqrt{n}}\omega^{-\frac{n-1}{2}}\sum_{k=0}^{n-1}|k\cdot \vec{\alpha }\rangle \end{array}$$

We have the following:

$$\begin{array}{cc} \; & QFT^{⊗n}\left(\frac{1}{\sqrt{n}}\omega^{-\frac{n-1}{2}}\sum_{k=0}^{n-1}|k\cdot \vec{\alpha }\rangle \right)=\omega^{-\frac{n-1}{2}}QFT^{⊗n}\left(\frac{1}{\sqrt{n}}\sum_{k=0}^{n-1}|k\cdot \vec{\alpha }\rangle \right)=\frac{1}{n^{\frac{n}{2}}}\omega^{-\frac{n-1}{2}}\sum_{\vec{k}\in F_{n}^{n}}a_{\vec{k}}|\vec{k}\rangle . \end{array}$$

By Lemma 1, $a_{\vec{k}}\neq 0$ iff $\sum_{l=1}^{n}k_{l}=0$. Therefore, the measurement result ${\left[s_{i}^{1},\ldots ,s_{i}^{n}\right]}^{T}$ must be $\vec{k}={\left[k_{1},\ldots ,k_{n}\right]}^{T}$ such that

$$\sum_{l=1}^{n}k_{l}=0=\prod_{j=0}^{n-1}j=\prod_{p=1}^{n}x_{i}^{p}.$$

   □

Now, we show that our protocol computes $GIP({\vec{x}}^{1},\ldots ,{\vec{x}}^{n})$ correctly. Since $s^{p}=\sum_{i}s_{i}^{p}$, by applying (6), we have the following:

$$\begin{array}{cc} \sum_{p}s^{p} & =\sum_{p}\sum_{i}s_{i}^{p}=\sum_{i}\sum_{p}s_{i}^{p}=\sum_{i}\prod_{p=1}^{n}x_{i}^{p}=GIP\left({\vec{x}}^{1},\ldots ,{\vec{x}}^{n}\right). \end{array}$$

## 4. Proposed Classical Protocol

We now consider purely classical protocols for our problem, i.e., ones that do not consider entanglement. At the top level, our classical scheme operates by communicating the “number” of different symbols that exist within each player’s vector. We show that this suffices for Alice to recover the function value.

More precisely, let $\beta_{k}^{p}$ be the number of “*k*” values in the vector of *p*-th player; recall that player *p* is assigned ${\vec{x}}_{p}=x_{1}^{p}\ldots x_{m}^{p}$. Note that $\sum_{k=0}^{n-1}\beta_{k}^{p}=m$.

**Theorem** **2.** *There exists a classical protocol for computing $GIP({\vec{x}}^{1},\ldots ,{\vec{x}}^{n})$ that uses ${\left(n-1\right)}^{2}\left(\log n^{2}\right)$ bits.*

In our protocol (see Algorithm 2), for each i=1,…,n−1, each player *p* transmits $\beta_{k}^{p}\;(\mathrm{mod}\;n^{2})$. Alice computes each $\beta_{0}^{p}\;(\mathrm{mod}\;n^{2})$ by using the fact that $\sum_{k=0}^{n-1}\beta_{k}^{p}=m$. Finally, Alice computes the value of the function by using $\left\{\beta_{k}^{p}\;(\mathrm{mod}\;n^{2})|k\in F_{n},p\in \left[n\right]\right\}$ For each player p∈{2,…,n}, *p* transmits $\left\{\beta_{1}^{p}\;(\mathrm{mod}\;n^{2}),\ldots ,\beta_{n-1}^{p}\;(\mathrm{mod}\;n^{2})\right\}$. The total number of bits transmitted is ${\left(n-1\right)}^{2}\left(\log n^{2}\right)$. The proof of the Theorem 2 appears in Appendix C.
**Algorithm 2:** Proposed Classical protocol.**for** player p∈{1,…n} **do**    **for** each $k\in F_{n}$ **do**        $\beta_{k}^{p}\leftarrow$ number of “*k*” values in $x_{1}^{p}\ldots x_{m}^{p}$        **if** *p* is not Alice **and** k≠0 **then**           *p* sends $\beta_{k}^{p}\;(\mathrm{mod}\;n^{2})$ to Alice        **end if**    **end for****end for****for** p∈{2,…n} **do**    Alice computes $\beta_{0}^{p}\;(\mathrm{mod}\;n^{2})$ by using    $\sum_{k=0}^{n-1}\beta_{k}^{p}=m$.**end for**$W\leftarrow \sum_{p=1}^{n}\sum_{k=1}^{n-1}k\cdot \beta_{k}^{p}+\frac{n^{2}-n}{2}\left(n-1\right)\sum_{p=1}^{n}\beta_{0}^{p}\;\left(\mathrm{mod}\;n^{2}\right)$$GIP({\vec{x}}^{1},\ldots ,{\vec{x}}^{n})=W/n$

## 5. Classical Communication Complexity Lower Bound

We now discuss a lower bound on the communication complexity of *any* classical protocol that demonstrates a strict separation between our proposed quantum protocol and any classical protocol. Analytically, this seems to be a rather hard problem, and we discuss it as an item for future work. We can show, however, the strict separation numerically using ILPs (see Section 5.1 below). In addition, we present an analytical argument below that demonstrates that for n=3, the communication complexity of any classical protocol is at least $2\log_{2}3$.

We assume that Alice, Bob, and Carol are given vectors ${\vec{x}}^{1}$, ${\vec{x}}^{2}$, and ${\vec{x}}^{3}$, respectively, each of length *m*. The promise (cf. Section 2.2) is equivalent to the following:

(9) $$\begin{array}{c} x_{j}^{1}+x_{j}^{2}+x_{j}^{3}=0,\;\mathrm{for}\;j=1,\ldots ,m. \end{array}$$

This implies that the GIP function in this case can be computed if we know any two out of ${\vec{x}}^{1},{\vec{x}}^{2}$, and ${\vec{x}}^{3}$. We assume that Carol labels her sequences (${\vec{x}}^{3}\in {\left\{0,1,2\right\}}^{m}$) with one of—at most—three possible labels. We denote this label by a mapping $\beta :{\left\{0,1,2\right\}}^{m}\to \left\{0,1,2\right\}$. Recall that Alice knows her sequence ${\vec{x}}^{1}$.

**Definition** **1.** *We define Bob’s confusion graph $G_{B}=\left(V_{B},E_{B}\right)$ as follows. The vertex set $V_{B}$ corresponds to the $3^{m}$ sequences ${\vec{x}}^{2}\in {\left\{0,1,2\right\}}^{m}$. The i-th such sequence is denoted by ${\vec{x}}^{2}\left[i\right]$ for $i=0,\ldots ,3^{m}-1$, with similar notations applied to the sequences for Alice and Carol.*

*There exists an edge $\left({\vec{x}}^{2}\left[i\right],{\vec{x}}^{2}\left[j\right]\right)\in E_{B}$, for i≠j if there exists an Alice sequence ${\vec{x}}^{1}\left[*\right]$ and Carol sequences ${\vec{x}}^{3}\left[a\right]$ and ${\vec{x}}^{3}\left[b\right]$, such that (i) $\beta \left({\vec{x}}^{3}\left[a\right]\right)=\beta \left({\vec{x}}^{3}\left[b\right]\right)$ (note that we allow a=b), and (ii) $GIP\left({\vec{x}}^{1}\left[*\right],{\vec{x}}^{2}\left[i\right],{\vec{x}}^{3}\left[a\right]\right)\neq GIP\left({\vec{x}}^{1}\left[*\right],{\vec{x}}^{2}\left[j\right],{\vec{x}}^{3}\left[b\right]\right)$.*

Note that if $\left({\vec{x}}^{2}\left[i\right],{\vec{x}}^{2}\left[j\right]\right)\in E_{B}$, Bob must assign different labels to ${\vec{x}}^{2}\left[i\right]$ and ${\vec{x}}^{2}\left[j\right]$; otherwise, Alice has no way to compute the function with zero error. The concept of the confusion graph dates back to the work of Shannon [22].

The main concept of the argument below is to show that there exists a triangle in $G_{B}$. This implies that Bob needs to use at least three labels for Alice to decode with zero error.

Since Carol uses, at most, three labels, the pigeon-hole principle dictates that there must be at least $3^{m-1}$ sequences that share the same Carol label. Let us denote this set by C.

**Claim** **1.** *There is a subset of two coordinates where all nine patterns* ${\left\{0,1,2\right\}}^{2}$ *appear within the sequences in* C.

**Proof.**  Suppose that *m* is even. Then, we can partition the coordinates as {1,2},{3,4},…,{m−1,m}. Let us arrange the sequences in C as rows; the number of rows is $\left|C\right|\geq 3^{m-1}$. Now, suppose that the projection onto any pair of coordinates has, at most, 8 representatives, then, the size of C can be, at most, $8^{m/2}$. Now, we have the following:

$$\begin{array}{cc} \frac{3^{m-1}}{8^{m/2}} & =\frac{1}{3}\times {\left(\frac{9}{8}\right)}^{m/2}\\ \; & >1, \end{array}$$

for large enough *m*. □

Without loss of generality, we assume that all nine patterns occur within the first two coordinates of C. We pick nine of such representatives from C and denote them as $z_{00},z_{01},z_{02},z_{10},\ldots ,z_{22}$; the subscripts correspond to the values on the first two coordinates.

Let us pick Alice’s sequence ${\vec{x}}^{1}\left[*\right]=\left[110\ldots 0\right]$. Corresponding to this ${\vec{x}}^{1}\left[*\right]$, for the Carol sequences $z_{00},\ldots ,z_{22}$, using the given promise, we can determine the corresponding Bob sequences $y_{00},\ldots ,y_{22}$. We note the following:

$$\begin{array}{cc} y_{00} & =[22-z_{00}\left(3:m\right)],\\ y_{01} & =[21-z_{01}\left(3:m\right)],\;\mathrm{and}\\ y_{11} & =[11-z_{11}\left(3:m\right)]. \end{array}$$

where $z_{00}\left(3:m\right)$ denotes the components of vector $z_{00}$ from index 3 onward (basically the MATLAB notation).

**Claim** **2.** *In Bob’s confusion graph,* $G_{B}$*, the sequences* $y_{00},y_{01},$ *and* $y_{11}$ *form a triangle.*

**Proof.**  We need to examine $GIP({\vec{x}}^{1}\left[*\right],y_{i},z_{i})$ for i=00,01,11. Since only the first two coordinates matter, given ${\vec{x}}^{1}\left[*\right]=\left[110\ldots 0\right]$, the corresponding evaluations are 0,1,2, which are pairwise different. □

This argument shows that Bob must use at least three labels for Alice to decode with zero error. By symmetry, Carol must also use three labels. To summarize, the communication complexity of any classical protocol is at least 2log3 bits.

**Remark** **2.** *It may be possible to use a variant of the above combinatorial argument to establish that the chromatic number of $G_{B}$ is strictly larger than three. However, this does not seem to follow in a straightforward manner.*

### 5.1. ILP Feasibility Problem for Classical Lower Bound

We now present a lower bound on the communication complexity of any deterministic classical protocol for our problem. To this end, we frame this as an integer linear programming problem (ILP) that can be solved numerically. The primary aim of the ILP is to establish a correspondence between each deterministic classical protocol and a feasible point within the ILP. Therefore, the feasibility of the ILP, which can be numerically verified, implies the existence of a deterministic classical protocol and vice versa (this correspondence between the ILP and classical protocols is valid only for the deterministic case. The ILP does not account for randomized protocols where players may have access to public and/or private randomness).

Suppose, for p∈{2,…,n}, the *p*-th player sends symbols (labels) in $\left[l^{p}\right]:=\left\{1,2,\ldots ,l^{p}\right\}$ for some large enough positive integer $l^{p}$. Let $c\in [l^{p}]$ and define $I_{{\vec{x}}^{p},c}\in \left\{0,1\right\}$ to be the indicator that the *p*-th player sends *c* when it has the vector ${\vec{x}}^{p}\in F_{n}^{m}$. As this mapping is unique, we have $\sum_{c\in [l^{p}]}I_{{\vec{x}}^{p},c}=1$. Furthermore, for a given set of vectors ${\vec{x}}^{p}$ for p∈{2,…,n}, if the *p*-th player sends label $c^{p}$, we have $\prod_{p=2}^{n}I_{{\vec{x}}^{p},c^{p}}=1$.

Consider two sets of vectors $\left\{{\vec{x}}^{p}\in F_{n}^{m}|p\in \left\{1,\ldots ,n\right\}\right\}$, $\left\{{\vec{z}}^{p}\in F_{n}^{m}|p\in \left\{1,\ldots ,n\right\}\right\}$. We denote the following:

$$\begin{array}{cc} \left({\vec{x}}^{1},\ldots ,{\vec{x}}^{n}\right) & {∼}_{GIP}\left({\vec{z}}^{1},\ldots ,{\vec{z}}^{n}\right) \end{array}$$

if the following conditions are satisfied.

1. Both $\left({\vec{x}}^{1},\ldots ,{\vec{x}}^{n}\right)$ and $\left({\vec{z}}^{1},\ldots ,{\vec{z}}^{n}\right)$ satisfy the promise (cf. Section 2.2).
2. ${\vec{x}}^{1}={\vec{z}}^{1}$.
3. $GIP\left({\vec{x}}^{1},\ldots ,{\vec{x}}^{n}\right)\neq GIP\left({\vec{z}}^{1},\ldots ,{\vec{z}}^{n}\right)$.

This definition applies to distinct inputs with the “same” Alice vector, but different function evaluations. It can be seen that—for two such distinct inputs—the symbols communicated by players 2 to *n* have to be distinct, otherwise, Alice has no way to decode in a zero-error fashion.

Our proposed ILP works with fixed $l^{p}$’s and a fixed value of *m*. Due to complexity reasons, *m* cannot be very large. However, if the ILP is infeasible for a given $l^{p}$ and a $\tilde{m}$, then our lower bound holds for arbitrary values $m\geq \tilde{m}$. Our lower bound would continue to hold even if Alice was provided the values $x_{\tilde{m}+1}^{p},\ldots ,x_{m}^{p}$ for all players p=2,…,n.

Consider the following 0−1 integer programming feasibility problemL

(10) $$\begin{array}{cc} \; & \min\;0\\ s.t.\; & p\in \left\{2,\ldots ,n\right\},c\in \left[l^{p}\right],{\vec{x}}^{p}\in F_{n}^{m},\\ \; & I_{{\vec{x}}^{p},c}\in \left\{0,1\right\},\\ \; & \sum_{c\in [l^{p}]}I_{{\vec{x}}^{p},c}=1,\forall {\vec{x}}^{p},\\ \;\sum_{c^{2}\in \left[l^{2}\right],\ldots ,c^{n}\in \left[l^{n}\right]} & |\prod_{p=2}^{n}I_{{\vec{x}}^{p},c^{p}}-\prod_{p=2}^{n}I_{{\vec{z}}^{p},c^{p}}|=2\\ \;\;\mathrm{for}\;\mathrm{all}\; & \left({\vec{x}}^{1},\ldots ,{\vec{x}}^{n}\right){∼}_{GIP}\left({\vec{z}}^{1},\ldots ,{\vec{z}}^{n}\right). \end{array}$$

The infeasibility of the above integer programming problem corresponds to a lower bound on the classical communication complexity. The proof of the following theorem appears in Appendix D.

**Theorem** **3.** *There exists a deterministic classical protocol computing GIP(·) where each player sends—at most—$l^{p}$ different labels for p∈{2,…,n} iff the above integer programming is feasible.*

**Remark** **3.** *The above integer program contains constraints that involve the product of variables and equality constraints with sums of absolute values. We show how these constraints can be linearized in Appendix E. The entire code for our ILP is available in this online repository [23].*

### 5.2. Numerical Experiments

In our numerical experiments, we considered an instance of the ILP involving n=3 players, namely Alice, Bob, and Carol. Let *m* represent the length of each vector, while $\left[l^{b}\right],\left[l^{c}\right]$ denote the sets of labels used by Bob and Carol, with $l^{b},l^{c}$ denoting the sizes of these sets.

We assume that Alice, Bob, and Carol are given vectors ${\vec{x}}^{1}$, ${\vec{x}}^{2}$, and ${\vec{x}}^{3}$, respectively, each of length *m*. In this case, the promise is given by the following: (9). It can be observed that swapping the vectors of Bob and Carol still satisfies the promise. Due to this inherent symmetry, a protocol with communication lengths $l^{b}=x$ and $l^{c}=y$ exhibits the same feasibility as one with $l^{b}=y$ and $l^{c}=x$. Consequently, for the ILP we can assume that $l^{b}\leq l^{c}$.

The experimental results under varying settings of $l^{b},l^{c},m$ are displayed in Table 1. For instance, it shows that when $l^{b}=1$ and $l^{c}=17$, the ILP is infeasible with m=3. This implies that for a feasible classical protocol, with $l^{b}=1$, we need at least log(18) bits to be transmitted from Carol. Similarly, the triplets $\left(m,l^{b},l^{c}\right)=\left(2,2,4\right)$ and (3,3,3) are infeasible. This implies that when $l^{b}$ equals 2 or 3, the sum rate is ≥min(log2+log5,log3+log4).

Recalling that our proposed protocol employs 2log(3) bits of communication, and by the fact that

$$\begin{array}{c} 2\log3<\min(\log18,\log3+\log4,\log2+\log5) \end{array}$$

we conclude that there is a strict separation between our quantum protocol and any classical protocol. We note here that we have expressed the communication complexity of both protocols in terms of bits by converting to base-2 logarithms. However, it is important to interpret the results, e.g., the quantum protocol is feasible if Bob and Carol use ternary communication (one of three possible symbols). Conversely, the classical protocol requires that at least one of Bob or Carol transmit one of four possible symbols. In this sense, the quantum protocol is strictly better.

## 6. Conclusions

We considered the communication complexity problem of the GIP function under a specific promise. We proposed a quantum protocol utilizing (n−1)log(n) bits and a classical protocol employing ${\left(n-1\right)}^{2}\left(\log n^{2}\right)$ bits. By establishing a connection between the integer linear programming feasibility problem and the existence of a classical protocol with a particular communication complexity, we were able to provide numerical evidence supporting the quantum advantage in our model’s communication complexity. The main limitation of our work is the absence of an analytical lower bound on the classical communication complexity of our problem.

It would be interesting to analytically investigate the quantum advantage in the asymptotic limit as *n* increases, and to consider promises where the quantum advantage can be analytically demonstrated, or other problems that also encompass the work in [5] as a special case.

## Figures and Tables

**Table 1:** Numerical results.

*m*	$l^{b}$	$l^{c}$	Feasibility
1	1	3	Feasible
3	1	17	Infeasible
2	2	4	Infeasible
3	3	3	Infeasible
2	3	4	Feasible
3	5	5	Feasible

## Data Availability

There is no significant data associated with the study. The corresponding author can be contacted if needed.

## Appendix A. Proof of Lemma 1

Recall that

$$\vec{\alpha }={\left[1,\ldots ,1\right]}^{T}.$$

The action of ${\mathrm{QFT}}^{⊗n}$ on $\frac{1}{\sqrt{n}}\sum_{j=0}^{n-1}\omega^{-xj}|j\cdot \vec{\alpha }\rangle$ is

$$\begin{array}{cc} \frac{1}{n^{\frac{n}{2}}}\sum_{\vec{k}\in F_{n}^{n}} & a_{\vec{k}}|\vec{k}\rangle =QFT^{⊗n}\left(\frac{1}{\sqrt{n}}\sum_{j=0}^{n-1}\omega^{-xj}|j\cdot \vec{\alpha }\rangle \right)\;=\frac{1}{n^{\frac{n}{2}}}\sum_{\vec{k}\in F_{n}^{n}}\left(\frac{1}{\sqrt{n}}\sum_{j=0}^{n-1}\omega^{-xj}\cdot \omega^{\langle \vec{k},j\cdot \vec{\alpha }\rangle }\right)|\vec{k}\rangle . \end{array}$$

Write $\vec{k}={\left[k_{1},\ldots ,k_{n}\right]}^{T}$. Therefore,

$$a_{\vec{k}}=\frac{1}{\sqrt{n}}\sum_{j=0}^{n-1}\omega^{-xj}\cdot \omega^{\langle \vec{k},j\cdot \vec{\alpha }\rangle }=\frac{1}{\sqrt{n}}\sum_{j=0}^{n-1}\omega^{-xj+j\sum_{l=1}^{n}k_{l}}.$$

When $\vec{k}$ satisfies $\sum_{l=1}^{n}k_{l}=x$, we have that $a_{\vec{k}}=\sqrt{n}\neq 0$. Otherwise, since $-x+\sum_{l=1}^{n}k_{l}\neq 0$ and *n* are prime, $1-\omega^{n(-x+\sum_{l=1}^{n}k_{l})}=0$ and $1-\omega^{-x+\sum_{l=1}^{n}k_{l}}\neq 0$. We have the following:

$$\begin{array}{c} \frac{1}{\sqrt{n}}\sum_{j=0}^{n-1}\omega^{-xj+j\sum_{l=1}^{n}k_{l}}=\frac{1}{\sqrt{n}}\sum_{j=0}^{n-1}\omega^{(-x+\sum_{l=1}^{n}k_{l})j}=\frac{1}{\sqrt{n}}\frac{1-\omega^{n(-x+\sum_{l=1}^{n}k_{l})}}{1-\omega^{-x+\sum_{l=1}^{n}k_{l}}}=0. \end{array}$$

## Appendix B. Derivation of Equations (7) and (8)

We derive Equation (7) by considering two cases. The first case occurs when ${\left[x_{i}^{1},\ldots ,x_{i}^{n}\right]}^{T}={\left[0,\ldots ,0\right]}^{T}$. Then, we have the following:

(A1) $$\begin{array}{cc} \; & P_{0}^{⊗n}\left(\frac{1}{\sqrt{n}}\sum_{k=0}^{n-1}|k\cdot \vec{\alpha }\rangle \right)\mapsto \frac{1}{\sqrt{n}}\omega^{-\frac{n(n-1)}{2}}|0\cdot \vec{\alpha }\rangle +\frac{1}{\sqrt{n}}\sum_{k=1}^{n-1}|k\cdot \vec{\alpha }\rangle =\frac{1}{\sqrt{n}}\sum_{k=0}^{n-1}|k\cdot \vec{\alpha }\rangle =\frac{1}{\sqrt{n}}\sum_{k=0}^{n-1}\omega^{-k\cdot 0}|k\cdot \vec{\alpha }\rangle . \end{array}$$

The second case occurs when ${\left[x_{i}^{1},\ldots ,x_{i}^{n}\right]}^{T}={\left[j,\ldots ,j\right]}^{T}$ with j≠0. Then, we have the following:

(A2) $$\begin{array}{c} P_{j}^{⊗n}\left(\frac{1}{\sqrt{n}}\sum_{k=0}^{n-1}|k\cdot \vec{\alpha }\rangle \right)\mapsto \frac{1}{\sqrt{n}}\sum_{k=0}^{n-1}\omega^{-n(\frac{1}{n}\left(kj\;\mathrm{mod}\;n\right))}|k\cdot \vec{\alpha }\rangle =\frac{1}{\sqrt{n}}\sum_{k=0}^{n-1}\omega^{-kj}|k\cdot \vec{\alpha }\rangle \end{array}$$

where the last equality holds since $\omega^{-kj}=\omega^{-(kj\;\mathrm{mod}\;n)}$. Thus, in this case, collectively, we can express the joint state after phase-shifting as $\frac{1}{\sqrt{n}}\sum_{k=0}^{n-1}\omega^{-kj}|k\cdot \vec{\alpha }\rangle$.

Now, we derive Equation (8). We have the following:

$$\begin{array}{cc} \; & P_{0}⊗\cdots ⊗P_{n-1}\left(\frac{1}{\sqrt{n}}\sum_{k=0}^{n-1}|k\cdot \vec{\alpha }\rangle \right)\mapsto \\ & \frac{1}{\sqrt{n}}\omega^{-\frac{n-1}{2}}|0\cdot \vec{\alpha }\rangle +\frac{1}{\sqrt{n}}\sum_{k=1}^{n-1}\omega^{-\sum_{j=0}^{n-1}\frac{1}{n}\left(kj\;\mathrm{mod}\;n\right)}|k\cdot \vec{\alpha }\rangle \\ \; & \overset{\left(a\right)}{=}\frac{1}{\sqrt{n}}\omega^{-\frac{n-1}{2}}|0\cdot \vec{\alpha }\rangle +\frac{1}{\sqrt{n}}\sum_{k=1}^{n-1}\omega^{-\frac{1}{n}\left(\sum_{j=0}^{n-1}j\right)}|k\cdot \vec{\alpha }\rangle \\ \; & =\frac{1}{\sqrt{n}}\omega^{-\frac{n-1}{2}}|0\cdot \vec{\alpha }\rangle +\frac{1}{\sqrt{n}}\sum_{k=1}^{n-1}\omega^{-\frac{1}{n}\left[\frac{n(n-1)}{2}\right]}|k\cdot \vec{\alpha }\rangle \\ & =\frac{1}{\sqrt{n}}\omega^{-\frac{n-1}{2}}\sum_{k=0}^{n-1}|k\cdot \vec{\alpha }\rangle \end{array}$$

where (a) follows from the fact that {kjmodn|j∈{0,…,n−1}}={0,…,n−1} for k≠0 (we emphasize that this statement only holds if *n* is prime). We have the following;

$$\begin{array}{cc} \; & QFT^{⊗n}\left(\frac{1}{\sqrt{n}}\omega^{-\frac{n-1}{2}}\sum_{k=0}^{n-1}|k\cdot \vec{\alpha }\rangle \right)=\frac{1}{\sqrt{n}}\omega^{-\frac{n-1}{2}}QFT^{⊗n}\left(\sum_{k=0}^{n-1}|k\cdot \vec{\alpha }\rangle \right)=\frac{1}{\sqrt{n}}\omega^{-\frac{n-1}{2}}\sum_{\vec{k}\in F_{n}^{n}}a_{\vec{k}}|\vec{k}\rangle . \end{array}$$

## Appendix C. Proof of Theorem 2

For $a,b\in F_{n}$, we define $M_{a,b}=\left\{i\in \left[m\right]|{\left[x_{i}^{1},\ldots ,x_{i}^{n}\right]}^{T}=a{\left[1,\ldots ,1\right]}^{T}+b{\left[0,1,\ldots ,n-1\right]}^{T}\right\}$ and $m_{a,b}=\left|M_{a,b}\right|$. Since the promise ensures that, for each i∈{1,…,m}, there exists $a,b\in F_{n}$ s.t. ${\left[x_{i}^{1},\ldots ,x_{i}^{n}\right]}^{T}=a{\left[1,\ldots ,1\right]}^{T}+b{\left[0,1,\ldots ,n-1\right]}^{T}\in F_{n}^{n}$, we have that $\left\{M_{a,b}|a,b\in F_{n}\right\}$ forms a partition of the set {1,…,m}.

When $i\in M_{a,0}$, i.e., ${\left[x_{i}^{1},\ldots ,x_{i}^{n}\right]}^{T}=a{\left[1,\ldots ,1\right]}^{T}$, we have the following:

(A3) $$\prod_{p=1}^{n}x_{i}^{p}=a^{n}=a.$$

Otherwise, we have $i\in M_{a,b}$ for some b≠0, so ${\left[x_{i}^{1},\ldots ,x_{i}^{n}\right]}^{T}=a{\left[1,\ldots ,1\right]}^{T}+b{\left[0,\ldots ,n-1\right]}^{T}$. Then, we have the following:

(A4) $$\prod_{p=1}^{n}x_{i}^{p}=\prod_{i=0}^{n-1}\left(a+i\cdot b\right)=0.$$

By (A3) and (A4), if $i\in M_{a,b}$, then $\prod_{p=1}^{n}x_{i}^{p}=\delta_{0b}\cdot a.$ Define

$$1\left(\left(x_{i}^{1},\ldots ,x_{i}^{n}\right),M_{a,b}\right)=\left\{\begin{array}{cc} 1, & i\in M_{a,b}\\ 0, & \mathrm{otherwise}, \end{array}\right.$$

i.e., it is the indicator of $i\in M_{a,b}$. Since $i\in M_{a,b}$ for exactly one choice of (a,b), we have the following:

(A5) $$\prod_{p=1}^{n}x_{i}^{p}=\delta_{0b}\cdot a=\sum_{a,b=0}^{n-1}\delta_{0b}\cdot a\cdot 1\left(\left(x_{i}^{1},\ldots ,x_{i}^{n}\right),M_{a,b}\right).$$

We have the following:

(A6) $$\begin{array}{cc} \; & \sum_{i=1}^{m}\prod_{p=1}^{n}x_{i}^{p}\\ \overset{\left(*\right)}{=} & \sum_{i=1}^{m}\sum_{a,b=0}^{n-1}a\cdot \delta_{0b}\cdot 1\left(\left(x_{i}^{1},\ldots ,x_{i}^{n}\right),M_{a,b}\right)\\ = & \sum_{a,b=0}^{n-1}\sum_{i=1}^{m}a\cdot \delta_{0b}\cdot 1\left(\left(x_{i}^{1},\ldots ,x_{i}^{n}\right),M_{a,b}\right)\\ = & \sum_{a,b=0}^{n-1}a\cdot \delta_{0b}\cdot m_{a,b}=\sum_{a=0}^{n-1}a\cdot m_{a,0}\;\left(\mathrm{mod}\;n\right). \end{array}$$

Here, (*) follows from (A5). Our next step is to show $\sum_{a=0}^{n-1}am_{a,0}=W/n\;(\mathrm{mod}\;n)$; *W* is defined in Algorithm 2.

Suppose $i\in M_{a,b}$, then $\left(x_{i}^{1},\ldots ,x_{i}^{n}\right)=a\left(1,\ldots ,1\right)+b\left(0,\ldots ,n-1\right)$. For the *p*-th player, a+(p−1)b is the value of the *i*-th coordinate of the vector ${\vec{x}}^{p}$. For a fixed $k\in F_{n}$, the set of (a,b) s.t. a+(p−1)b=k is {(k,0),(k+p−1,−1),(k+2p−2,−2),…,(k+(n−1)(p−1),−(n−1)}. We have the following: $\forall p\in \left\{1,\ldots ,n\right\},k\in F_{n},$

(A7) $$\beta_{k}^{p}=\sum_{i=0}^{n-1}m_{k+i(p-1),-i}.$$

Consider $\sum_{i=0}^{n-1}\sum_{p=1}^{n}m_{k+i(p-1),-i}$. When i=0, $m_{k+i(p-1),-i}$ is counted *n* times. Denote S={(0,0),…,(n−1,0)}. For arbitrary ${\left[x,y\right]}^{T}\in F_{n}^{2}-S$, the equation

$$\begin{array}{cc} \left[\begin{array}{c} k+i(p-1)\\ -i \end{array}\right]\; & =\left[\begin{array}{c} x\\ y \end{array}\right] \end{array}$$

has a unique solution given by the following:

$$\begin{array}{cc} i & =-y,\;\mathrm{and}\\ p & =\frac{y-x+k}{y}. \end{array}$$

Thus, we have the following:

$$F_{n}^{2}-S=\{\left(k+i\left(p-1\right),-i|p\in \left\{1,\ldots ,n\right\},i\in \left\{1,\ldots ,n-1\right\}\right\}.$$

Therefore, when i≠0, $m_{k+i(p-1),-i}$ is counted exactly once. We have the following:

(A8) $$\begin{array}{cc} \; & \sum_{p=1}^{n}\sum_{i=0}^{n-1}m_{k+i(p-1),-i}\\ = & \sum_{p=1}^{n}\sum_{i=1}^{n-1}m_{k+i(p-1),-i}+\sum_{p=1}^{n}m_{k+0(p-1),0}\\ = & \sum_{a,b\in F_{n}^{2}-S}m_{a,b}+n\cdot m_{k,0}. \end{array}$$

Now, we have the following:

$$\begin{array}{cc} \sum_{p=1}^{n}\sum_{k=1}^{n-1}k\cdot \beta_{k}^{p}\overset{\left(A7\right)}{=} & \sum_{k=1}^{n-1}k\sum_{p=1}^{n}\sum_{i=0}^{n-1}m_{k+i\cdot (p-1),-i}\\ \overset{\left(A8\right)}{=} & \sum_{k=1}^{n-1}k\left[\sum_{a,b\in F_{n}^{2}-S}m_{a,b}+n\cdot m_{k,0}\right]\\ = & \frac{n^{2}-n}{2}\sum_{a,b\in F_{n}^{2}-S}m_{a,b}+n\sum_{k=1}^{n-1}k\cdot m_{k,0}\\ \mathrm{and}\;\sum_{p=1}^{n}\beta_{0}^{p}\overset{\left(A7\right)}{=} & \sum_{p=1}^{n}\sum_{i=0}^{n-1}m_{i\cdot (p-1),-i}\overset{\left(A8\right)}{=}\sum_{a,b\in F_{n}^{2}-S}m_{a,b}+n\cdot m_{0,0}. \end{array}$$

Note n>2 is prime, so n−1 is divisible by 2. We have the following:

$$\begin{array}{cc} W= & \sum_{p=1}^{n}\sum_{k=1}^{n-1}k\cdot \beta_{k}^{p}+\frac{n^{2}-n}{2}\cdot \left(n-1\right)\sum_{p=1}^{n}\beta_{0}^{p}\\ = & \frac{n^{2}-n}{2}\sum_{a,b\in F_{n}^{2}-S}m_{a,b}+n\sum_{k=1}^{n-1}k\cdot m_{k,0}+\frac{n^{2}-n}{2}\cdot \left(n-1\right)\left[\sum_{a,b\in F_{n}^{2}-S}m_{a,b}+n\cdot m_{0,0}\right]\\ = & n\sum_{k=1}^{n-1}k\cdot m_{k,0}+n^{2}\cdot \frac{\left(n-1\right)}{2}\sum_{a,b\in F_{n}^{2}-S}m_{a,b}+n^{2}\cdot \frac{{\left(n-1\right)}^{2}}{2}\cdot m_{0,0}\\ = & n\sum_{k=0}^{n-1}k\cdot m_{k,0}\;\left(\mathrm{mod}\;n^{2}\right). \end{array}$$

When we divide both sides by *n*, we obtain $W/n=\sum_{k=1}^{n-1}km_{k,0}\;\left(\mathrm{mod}\;n\right)$. We are now done because of (A6).

## Appendix D. Proof of Theorem 3

Suppose we have a protocol that computes the function with zero error. Our protocol is deterministic, so for each ${\vec{x}}^{p}\in F_{n}^{m}$, it associates exactly one label $\hat{c}$ such that the *p*-th player sends $\hat{c}$ if his vector is ${\vec{x}}^{p}$. We set $I_{{\vec{x}}^{p},\hat{c}}=1$ and $I_{{\vec{x}}^{p},c}=0$ for all $c\neq \hat{c}.$ Therefore, $I_{{\vec{x}}^{p},c}\in \left\{0,1\right\}$ and $\sum_{c\in [l]}I_{{\vec{x}}^{p},c}=1$ are satisfied for all choices of ${\vec{x}}^{p},c$.

Next, suppose we have that $\left({\vec{x}}^{1},\ldots ,{\vec{x}}^{n}\right){∼}_{GIP}\left({\vec{z}}^{1},\ldots ,{\vec{z}}^{n}\right)$. Furthermore, assume that the *p*-th player sends $s^{p}/t^{p}$ for ${\vec{x}}^{p}/{\vec{z}}^{p}$ for p=2,…,n. This implies that $\prod_{p=2}^{n}I_{{\vec{x}}^{p},s^{p}}=1$ and that $\prod_{p=2}^{n}I_{{\vec{z}}^{p},t^{p}}=1$. In addition, note that ∃p, such that $s^{p}\neq t^{p}$ for these sequences, the symbols transmitted from users 2,…,n have to be distinct.

Thus, we have the following:

$$\begin{array}{c} 1\;=\;|\prod_{p=2}^{n}I_{{\vec{x}}^{p},s^{p}}-\prod_{p=2}^{n}I_{{\vec{z}}^{p},s^{p}}\left|=\right|\prod_{p=2}^{n}I_{{\vec{x}}^{p},t^{p}}-\prod_{p=2}^{n}I_{{\vec{z}}^{p},t^{p}}| \end{array}$$

and, consequently, we have the following:

$$\begin{array}{cc} \; & \sum_{c_{2}\in \left[l^{2}\right],\ldots ,c_{n}\in \left[l^{n}\right]}\left|\prod_{p=2}^{n}I_{{\vec{x}}^{p},c^{p}}-\prod_{p=2}^{n}I_{{\vec{z}}^{p},c^{p}}\right|=\\ \; & |\prod_{p=2}^{n}I_{{\vec{x}}^{p},{\hat{s}}^{p}}-\prod_{p=2}^{n}I_{{\vec{z}}^{p},{\hat{s}}^{p}}\left|+\right|\prod_{p=2}^{n}I_{{\vec{x}}^{p},{\hat{t}}^{p}}-\prod_{p=2}^{n}I_{{\vec{z}}^{p},{\hat{t}}^{p}}|=2. \end{array}$$

Therefore, the third constraint is satisfied.

Conversely, if $\left\{I_{{\vec{x}}^{p},c}|{\vec{x}}^{p}\in F_{n}^{m},c\in \left[l^{p}\right]\right\}$ satisfies the constraints of the ILP, then we construct a classical protocol as follows. Suppose the *p*-th player has vector $\vec{x^{p}}$ for p∈{1,…,n}. Since there exists exactly one $s^{p}\in \left[l^{p}\right]$ s.t. $I_{x^{p},s^{p}}=1$, then *p* sends $s^{p}$ to Alice for p∈{2,…,n}. When Alice receives the symbols $s^{p},p=2,\ldots ,n$ from the other players, she picks arbitrary ${\left\{{\vec{y}}^{p}\in F_{n}^{m}\right\}}_{i=2}^{n}$ s.t. $\left({\vec{x}}^{1},{\vec{y}}^{2},\ldots ,{\vec{y}}^{n}\right)$ satisfies the promise and $\forall p\in \left\{2,\ldots ,n\right\},I_{{\vec{y}}^{p},s^{p}}=1$. Then, she outputs $f({\vec{x}}^{1},{\vec{y}}^{2},\ldots ,{\vec{y}}^{n})$. In what follows, we show that

$$GIP\left({\vec{x}}^{1},{\vec{y}}^{2},\ldots ,{\vec{y}}^{n}\right)=GIP\left({\vec{x}}^{1},{\vec{x}}^{2},\ldots ,{\vec{x}}^{n}\right).$$

To see this, assume otherwise. Then, we have the following: $GIP\left({\vec{x}}^{1},{\vec{y}}^{2},\ldots ,{\vec{y}}^{n}\right)\neq GIP\left({\vec{x}}^{1},{\vec{x}}^{2},\ldots ,{\vec{x}}^{n}\right).$ We have the following:

$$\left({\vec{x}}^{1},{\vec{x}}^{2},\ldots ,{\vec{x}}^{n}\right){∼}_{GIP}\left({\vec{x}}^{1},{\vec{y}}^{2},\ldots ,{\vec{y}}^{n}\right).$$

Owing to the third constraint, we have the following:

$$\sum_{c^{2},\ldots ,c^{n}\in \left[l\right]}\left|\prod_{p=2}^{n}I_{{\vec{x}}^{p},c^{p}}-\prod_{p=2}^{n}I_{{\vec{y}}^{p},c^{p}}\right|=2.$$

However, we have that $I_{{\vec{x}}^{i},s^{i}}=I_{{\vec{y}}^{i},s^{i}}=1$ for all i∈{2,…,n}. By the first and second constraints, we have that $I_{{\vec{x}}^{i},c^{i}}=I_{{\vec{y}}^{i},c^{i}}=0$ for all i∈{2,…,n} and $c^{i}\neq s^{i}$. Therefore,

$$\sum_{c^{2},\ldots ,c^{n}\in \left[l\right]}\left|\prod_{p=2}^{n}I_{{\vec{x}}^{p},c^{p}}-\prod_{p=2}^{n}I_{{\vec{y}}^{p},c^{p}}\right|=0.$$

This gives the desired direction.

## Appendix E. Linearizing Constraints in the Integer Programming Problem

One issue with the optimization problem in (10) is that the third constraint has multiple absolute values and products of variables. Here, we transform the constraints and add extra variables in (10) to obtain the desired ILP.

Our first step is to introduce auxiliary 0–1 variables that correspond to the products of other 0–1 variables. For instance, it can be verified that we can handle $\prod_{i=1}^{k}x_{i}=x^{\prime }$ as follows:

(A9) $$\begin{array}{cc} x^{\prime } & \leq x_{i},\;\mathrm{for}\;i=1,\ldots ,k \end{array}$$

(A10) $$\begin{array}{cc} x^{\prime } & \geq \sum_{i=1}^{k}x_{i}-\left(k-1\right). \end{array}$$

As a first step, we introduce such auxiliary variables for all terms that involve products of our indicator function in (10).

Following this step, we are left with handling constraints that involve sums of absolute values of differences. For this step, we show how to replace each absolute value difference with another auxiliary variable. In particular, we can replace |x−y| by *z* as follows:

$$\begin{array}{c} \left|x-y\right|={\left|x-y\right|}^{2}=x^{2}+y^{2}-2xy=x+y-2xy \end{array}$$

where the last step follows from the fact that the variables are of type 0–1. The product term 2xy can be linearized as described previously. Following these steps, all constraints in the integer programming problem are linear.
